# Development and validation of the Safe Sleep Calculator to assess risk of sudden unexpected death in infancy

**DOI:** 10.1038/s41598-022-10201-3

**Published:** 2022-04-12

**Authors:** C. G. McIntosh, J. M. D. Thompson, K. Leech, R. Carpenter, E. A. Mitchell

**Affiliations:** 1 Department of Paediatrics, Child and Youth Health, Faculty of Medical and Health Sciences, The University of Auckland, Auckland, New Zealand; 2 Primary and Integrated Care, Counties Manukau Health District Health Board, Auckland, New Zealand; 3 Procon Ltd., Auckland, New Zealand; 4 Department of Medical Statistics, London School of Hygiene & Tropical Medicine, London, UK

**Keywords:** Software, Diagnosis, Disease prevention, Patient education, Paediatrics, Paediatric research, Epidemiology, Paediatric research, Translational research, Risk factors

## Abstract

We describe the development and validation of a Sudden Unexpected Death in Infancy (SUDI) risk assessment clinical tool. An initial SUDI risk assessment algorithm was developed from an individual participant data meta-analysis of five international SIDS/SUDI case–control studies. The algorithm was translated into a clinical web tool called the Safe Sleep Calculator, which was tested at the routine infant 6-week check-up in primary care clinics in New Zealand. Evidence was gathered through mixed-methods research to inform the revision of the algorithm and the clinical tool. The revised algorithm performance was validated on a new contemporary New Zealand SUDI case–control study dataset and the pilot population data set. The area under the Receiver Operator Characteristic (ROC) curve is 0.89, with a sensitivity of 83.0% and a specificity of 80.9% in the NZ infant population when 0.3 per 1000 live births or more risk is used to define ‘at higher risk’. The Safe Sleep Calculator SUDI risk assessment tool provides individualized evidence-based specific SUDI prevention advice for every infant and enables the concentration of additional SUDI prevention efforts and resource for infants at higher risk.

## Introduction

Sudden Unexpected Death in Infancy (SUDI) remains the leading category of death in babies in high-income countries contributing one-third to a half of all post-neonatal mortality^1^. SUDI includes deaths certified as Sudden Infant Death Syndrome (SIDS; ICD R95), ill-defined and unknown cause of mortality (R99) and accidental suffocation and strangulation in bed (W75). While the causes of SUDI remain a subject of research, International case–control studies have consistently identified contributory risk and protective factors for SUDI and SIDS that have remained consistent over time and in different countries^2^. Broad population health messages based on the epidemiology of SUDI/SIDS have been effective for the prevention of SUDI/SIDS. For example, the ‘Back to Sleep’ campaign in the early 1990s resulted in a rapid sustained reduction in SIDS in New Zealand and internationally. However, such an approach has not been effective enough for all groups. Although in New Zealand whilst Māori SUDI rates have declined, Māori infants remain nearly seven times, and Pacific Peoples infants nearly four times more likely to die of SUDI compared to Non-Māori/Non-Pacific (predominantly European) infants in New Zealand^3,4^.

Information sharing between parents and clinicians about the risks of bedsharing for an individual baby is important because bedsharing markedly increases the risk for some babies but is a much lesser risk for others. Clinicians require knowledge to move from rule-based dialogue to informed conversations with parents. This is particularly important for Māori and Pacific Peoples families for whom rates of maternal smoking in pregnancy are higher than for other population groups, and bedsharing with infants is also a culturally valued and sometimes necessary behaviour due to the effects of poverty induced crowded housing. A SUDI risk assessment tool may help to support effective conversations with parents about the choices they make to lower SUDI risk and prompt specific targeted resources and support to reduce SUDI risk.

Using scoring systems for SUDI/SIDS risk is not a new concept with a number developed from the 1970s through to recent years, some of which have been implemented and used to target SUDI/SIDS intervention programs^5–8^. Early scoring systems were hampered by a lack of sensitivity and reproducibility of results when used in different geographic areas of the same country, and between countries^9,10^. Nevertheless, the areas in which interventions were implemented for those at higher risk did observe a reduction in SIDS^11^. It is now reasonable to revisit the concept of SUDI risk assessment as infant sleep position and the interaction between maternal smoking in pregnancy and bedsharing were not a part of these early SIDS scoring systems and they have since proven to be important modifiable SUDI risk factors.

In 2013 Carpenter et. al. published the results of an individual participant data (IPD) meta-analysis of five international case–control studies (The European Concerted Action on SIDS 1992–1996, the Scottish 1996–2000, the New Zealand 1987–1990, the Irish 1994–2003 and the German GeSID 1998–2001) with a combined data set of 1472 cases and 4679 controls^12^. Carpenter, at the prompting of Mitchell, developed a SUDI risk assessment algorithm (see supplementary information) based on the modelling from the IPD meta-analysis that accounted for 16 contributory factors and interactions, and Mitchell then had a software application (App) developed with the algorithm called the Cure Kids SUDI Risk Assessment App^13^.

We hypothesized that we could achieve a new approach to SUDI risk assessment in New Zealand using the Carpenter algorithm by building this into a clinical tool, which would enable an evidence-based objective SUDI assessment and tailored SUDI protection education and supportive care. We aimed to develop, pilot, and refine a SUDI risk assessment web-based tool for clinical use.

## Methods

**Figure 1:**
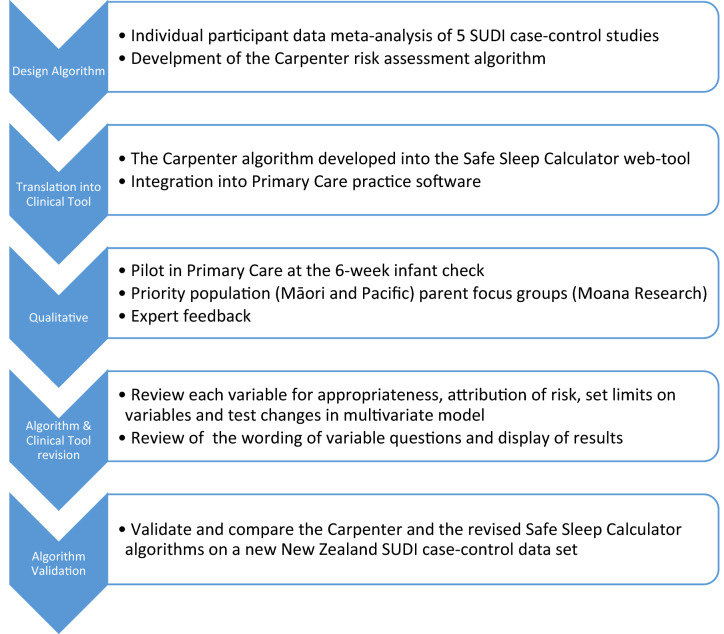
Study design showing the stages of translational research to develop, test and revise the Safe Sleep Calculator algorithm and clinical tool.

A mixed-methods translational research design was used because this fitted with developing the evidence base of epidemiological SUDI/SIDS research into a clinically useful SUDI risk assessment tool^14^. This enabled a quantitively derived algorithm to be translated through stakeholder engagement into a useful clinical tool, and then the revised algorithm performance to be validated in a new SUDI case–control dataset. The qualitative research that is relevant to the questions, structure, and limits of the variables in the algorithm when in clinical use is discussed in this article. Acceptability of the SUDI risk assessment approach for Māori and Pacific parents and families was completed by Māori and Pacific health researchers led by Moana Research^15^. Figure 1 shows the stages of progression of the study.

Ethics approval for this study was granted by the New Zealand Health and Disability Ethics Committee (HDEC) approval HDEC 16/NTB/53. The study is registered under Clinical Trial Registration ACTRN12616000355471, 18/03/2016. This research was performed in accordance with the Declaration of Helsinki, and per the methods reviewed and approved by HDEC, the participating Primary Health care Organisations and Primary Care practices, according to the relevant guidelines and regulations of the organisations.

### Translation of SUDI risk algorithm into a clinical tool

The Carpenter algorithm was developed into a web-based tool called the Safe Sleep Calculator (SSC) by the authors. It consists of questions relating to the 16 variables in Carpenter’s algorithm and returns an absolute risk score and information about the risk reduction from changing behaviours. The web tool was designed to be accessible as an integrated web form within the Practice Management System’s (PMS) in Primary Care. When used within the PMS the form is shorter because it sources some patient information from the PMS. The assessment result is saved to the clinical record. The web tool displays a link to an online SUDI prevention care clinical pathway that provides additional information about services that support families with SUDI prevention care, including access to an appropriate baby bed which is provided to families with SUDI prevention education.

### Clinical pilot

The Safe Sleep Calculator tool was piloted at the routine infant 6-week assessment in nine primary care general practice clinics in New Zealand from July 2016 to July 2017. Primary Care use was chosen as a pilot clinical situation because web-tool integration is comparatively simple in Primary Care in New Zealand. It enabled trialling in a variety of practice settings and population groups. Locality approvals were obtained from the Primary Health Care Organisations (PHO’s) and participating practices.

Electronically documented informed verbal consent was obtained from the mother, parent or guardian of the infant, as required for participants under 18 years of age, for the use of the Safe Sleep Calculator as part of the assessment of the infant. The consent covered the assessment and the use of the health information collected by the Safe Sleep Calculator web tool to be used by the research team in a research database for reporting in an aggregate and non-identifiable form. Informed consent was supported by providing both the information about the study and the electronic documentation consent tick box within the SUDI assessment digital form, and accessible within the health record of the infant. This form of consent was considered appropriate by HDEC who approved this study.

Seven practices met the criteria of reasonable use (a minimum of 4 months pilot and at least five or more Safe Sleep Calculator assessments) to participate in qualitative feedback on the tool. Informed written consent was obtained from each practitioner for focus group participation. Focus group discussions were recorded, then transcribed and analysed. Additional written feedback was provided on the Safe Sleep Calculator web tool by experts in SUDI prevention in New Zealand. This paper reports on qualitative feedback which directly impacts the algorithm. A further paper will describe the qualitative evaluation of implementing the Safe Sleep Calculator in Primary Care.

### Review of the webtool and internal validation of the algorithm

Each contributory variable in the original Safe Sleep Calculator clinical tool was reviewed based on the clinical pilot qualitative feedback. The specific variables of the Carpenter algorithm and the changes implemented to form the new revised Safe Sleep Calculator algorithm are described in Table 1 with further explanatory commentary in the following sections.

**Table 1:** Comparison of the Carpenter algorithm and the revised Safe Sleep Calculator algorithm.

Algorithm variables	Responses		Carpenter algorithm		Safe Sleep Calculator algorithm		Rationale
Baseline population risk (rate per 1000 live births)	n/a		0.50		0.73		The New Zealand population baseline risk. Adjust this variable for use in other countries
Maternal age (years)	15		6.31		4.96		Limits ≤ 18 = 18 years, ≥ 30 = 30 years Modelling changed to reduce the exaggerated effects of young maternal age Set neutral risk at 30 years
	18		3.33		4.96		
	20		2.27		3.47		
	25		1.00		1.66		
	30		0.54		1.00		
	40		0.30		1.00		
Birth order	1		1.71		1.50		Based on univariate CDC data limits ≥ 6 = 6, minor change in OR due to revision of other variables in the multivariable model
	2		2.94		2.24		
	3		5.04		3.35		
	4		8.64		5.01		
	5		14.82		11.22		
	6		25.40		16.79		
	7+		43.55		16.79		
Mother partner status	Partnered		1.00		1.00		Minor change due to revision of other variables in the multivariable model
	Single		1.80		1.40		
Breastfeeding	Breastfeeding		1.00		1.00		Minor change due to revision of other variables in the multivariable model
	Artificial feeding		1.47		1.66		
Infant sex	Female		1.00		1.00		Minor change due to revision of other variables in the multivariable model
	Male		1.57		1.40		
Infant ethnicity	Higher risk		1.31		–		Not used for risk assessment in revised clinical tool
	Population groups with higher SUDI rate		–		(1.31)		Used for prioritisation for support purposes only
Infant birthweight (grams)	1000		6.09		2.95		Maximum and minimum limits ≤ 1800 g = 1800 g ≥ 3800 g = 3800 g
	1500		3.88		2.95		
	1800		2.96		2.95		
	2000		2.47		2.46		
	2500		1.57		1.57		
	3000		1.00		1.00		
	3500		0.64		0.64		
	3800		0.49		0.49		
	4000		0.41		0.49		
	4500		0.26		0.49		
Infant multiple	Singleton		1.00		1.00		No change
	Multiple		2.40		2.40		
Sleep room	Same as parent		1.00		1.00		Minor change due to revision of other variables in the multivariable model
	Separate room		2.41		1.80		
Position placed to sleep and age of infant and bedsharing			Not bedsharing	Bedsharing	Not bed sharing	Bedsharing	Removed ≥ 3 months assessment range age effect because assessment is most likely to occur in the first few weeks from birth. In the revised algorithm the OR for sleep position is the same for bedsharing and non-bedsharing
Back	< 3-months		1.00	}2.82	1.00	1.00	
Side			1.51		1.26	1.26	
Prone			11.06		4.97	4.97	
Back	≥ 3-months		1.00	1.00	–	–	
Side			1.39	0.32	–	–	
Prone			7.90	2.11	–	–	
Tobacco smoking and bedsharing	Maternal smoking		1.40	5.38	1.46	15.12	Removal of the interaction of maternal drug use in the bedsharing scenario increases the smoking effect in the revised multivariate model. Bedsharing risk remains in a young infant (< 3 months) if neither parent smokes
	Other parent smoking		1.06	2.12	1.23	7.36	
	Both parents smoke		2.64	7.07	3.10	26.13	
	Neither smoke		–	–	1.00	3.00	
Maternal drug use	Any illicit drug use		12.15	94.91	–	–	Removal of the interaction with bedsharing, attenuation of risk, and separation of cannabis from other illicit drugs
	Cannabis use		–	–	2.00	2.00	
	Other illicit drug use		–	–	2.00	2.00	
Maternal alcohol use	Days/week in past 2 weeks	1 day	1.25	1.38			Removed frequency of alcohol consumption and attenuation of risk. An increased risk associated with bedsharing
		3 days	1.97	2.64			
		7 days	4.85	9.64			
	≥ 2 standard drinks in 24 h at any time		–	–	2.00	4.00	

CDC = Centers of Disease Control and Prevention, OR = Odds Ratio (see supplementary information for the revised algorithm).

### Localising baseline population risk

Carpenter’s algorithm comprises 16 contributory variables which act upon the baseline population SUDI rate of 0.5 per 1000 live births. For New Zealand use the baseline rate has been redefined at 0.73 per 1000 live births^16^. Any use of the algorithm in other countries should be accompanied by adjustment of baseline to the local SUDI whole population rate.

### Variables with minimal change

Mother not married or partnered, male infant, twin/multiple, not breastfeeding and baby sleeping in a separate room to parent are all binary variables and independent risk factors. The odds ratios associated with these factors in the revised algorithm are slightly different from the Carpenter algorithm due to changes made to other variables in the revised multivariable model (see Table 1).

### Introducing limits on variables

**Figure 2:**
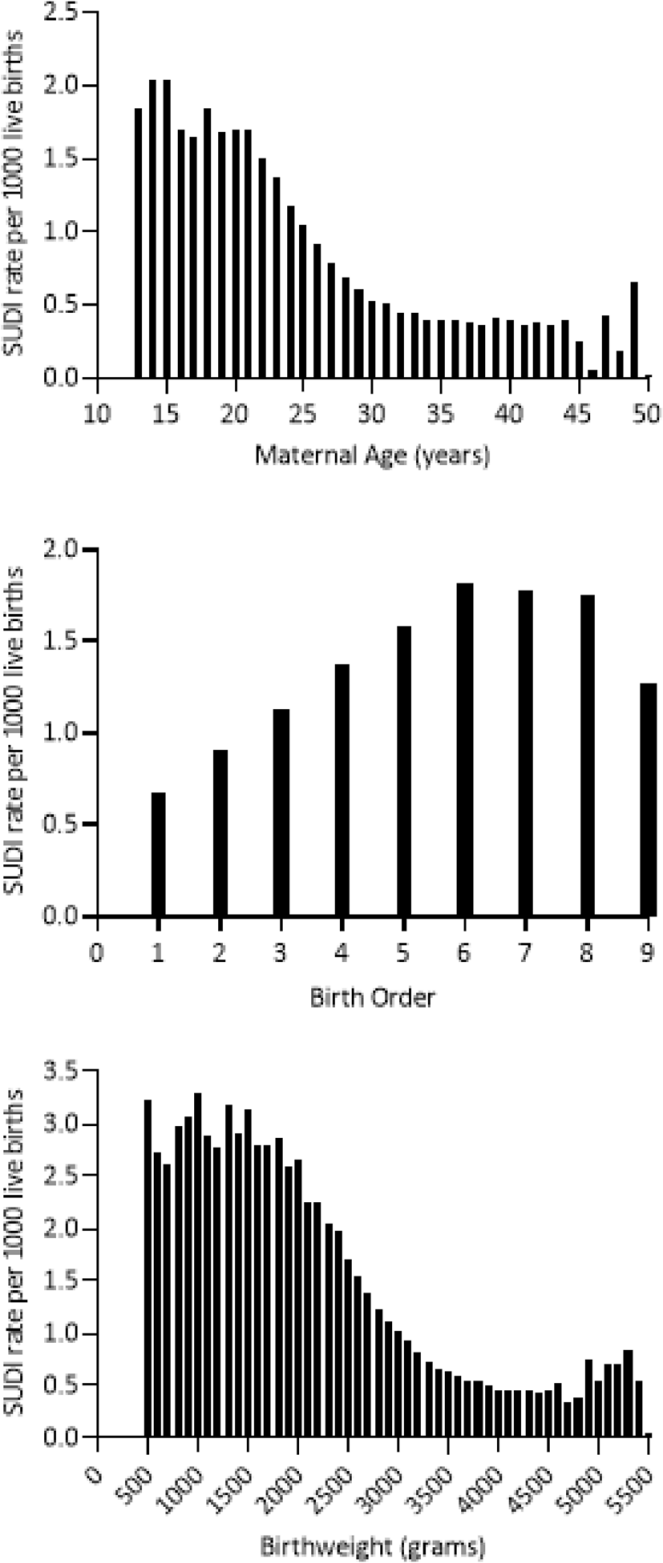
Centres of Disease Control and Prevention univariate data Sudden unexpected infant death (SUID) per 1000 live births 2003–2013 for maternal age, birthweight, and live birth order (SUID is the USA equivalent to SUDI).

During clinical use, it became evident that limits needed to be set on continuous variables because extreme values influenced the overall calculated risk substantially and probably unrealistically. For example, in the Carpenter algorithm, there was an inverse linear relationship between maternal age and risk of SUDI, thus the risk calculated for an infant of a young mother was inevitably very high and modification of other risks made comparatively little impact on overall risk. Practitioners reported that it was not helpful to present young mothers with a high unmodifiable risk because it frightened them, and they might already be doing everything they could to reduce SUDI risk. Hence the decision was made to limit the risk associated with young maternal age. This was supported by Centers for Disease Control (CDC) data that showed the SUDI rates did not increase further with very young maternal age (Fig. 2). Conversely, for mothers over the age of 30 years, the Carpenter modeling produced an increasingly protective effect for older mothers which attenuated the effect of other risk factors. While recognizing the limitations of univariable data, the CDC Infant mortality data 2003–2013 suggested that it was reasonable to set the upper limit of maternal age effect at 30 years for risk calculation (Fig. 2)^17^.

Carpenter’s algorithm calculates a linear risk relationship for birth order with a limit at seven. Examination of the CDC Cohort Linked Birth/Infant Death Data Set suggests that the risk plateaus beyond a birth order of six (Fig. 2), and so the limit has been revised down^17^.

Modelling of birthweight in a linear manner has remained the same between the Carpenter and the Safe Sleep Calculator algorithm. However, the CDC data supports limits being placed at the ends of the distribution (Fig. 2). Consistent with the CDC data, and recognizing prematurity would otherwise contribute to high unmodifiable risk, the revised algorithm adds no additional risk for birthweights under 1800 g, nor diminishing of risk for birthweights over 3800 g^17^. Note that SUDI risk associated with prematurity at birth and small for gestational age is accounted for by birthweight, similarly to Smith and White who found no additional predictive benefit of separating these factors^18^.

### Infant age, bedsharing, and sleep position

The Carpenter algorithm dichotomizes infant age into less than 3 months and greater than 3 months of age and has no additional risk accorded for non-back sleep position during bedsharing until 3 months old (Table 1). The clinical users reported that the Safe Sleep Calculator is of most use in the antenatal period and the first few weeks from birth and thereafter the risk for an infant is unlikely to be recalculated, and infant care behaviours will generally be established. As SUDI risk reduces with increasing age and most assessment events will occur close to birth, the revised algorithm has removed the effect of age and provides the risk over the baby’s first year of life.

Bedsharing and infant sleep position are independent choices parents make for sleeping their infants. The Carpenter algorithm attributed equal risk for all sleep positions for bedsharing infants under 3 months of age and therefore the resulting risk assessment did not differentiate risk for sleep position in this group and missed the opportunity to show the protective effect of back sleeping. In the revised algorithm we have chosen to separate sleep position from bedsharing risk because this provides clarity of SUDI prevention messaging. We have assigned an OR for bedsharing of 3.0 (Table 1) appropriate for a tool most likely to be used early in an infant's life, and consistent with the range findings of Carpenter et. al. who found an adjusted OR of 2.7 (95% CI 1.4–5.3) for all ages and 5.1 (95% CI 2.3–11.4) for infants less than 3-months with no other risk factors^12^.

### Revising risk attribution for alcohol and drugs and interaction with bedsharing

The IPD meta-analysis from which the Carpenter algorithm was derived had significant amounts of imputed data for maternal alcohol (61.3%) and maternal drug use (60.5%) because the participating centres collected these data in a variety of formats, had a lot of missing data, or the data was not collected at all^12^. The Carpenter et. al. IPD meta-analysis calculated an adjusted odds ratio of 4.8 (95% CI 2.6–8.9) for maternal alcohol consumption in the past 24 h and 11.5 (95% CI 2.2–59.5) for any maternal drug use since birth in a non-bedsharing infant^12^. Carpenter took the approach in the algorithm of recording maternal alcohol use as the number of days per week with two or more standard drinks, and accumulated risk depending on the number of days of drinking with an escalation of risk in the bedsharing situation. This approach is potentially problematic if the mother occasionally drinks a large amount of alcohol and it misses the opportunity of assessment of harm to both mother and infant. Therefore, the revised Safe Sleep Calculator algorithm introduces the validated World Health Organisation Alcohol Use Disorders Identification Test of consumption (AUDIT-C) alcohol screening to address these concerns^19^. Data internationally is variable in the definition of alcohol use in quantity and frequency in SUDI/SIDS studies, and multivariable odds ratios vary with evidence of an interaction between alcohol and bedsharing in some studies and not others^20–23^. Because of this uncertainty and the problem of presenting immovable risk in clinical use, we have assigned an OR of 2.0 for two or more standard drinks of alcohol on any day (24 h) in the past month and attributed an escalated OR of 4.0 if the mother also bedshares. The setting of the ORs at these levels is likely to be conservative and is at the lower end of the confidence interval in the IPD meta-analysis^12^. However, setting these ORs for alcohol performs well in the revised Safe Sleep Calculator model external validation in a complete data set (Table 2) and taking an AUDIT-C approach achieves meaningful screening for alcohol harm for both mother and baby and additional assessment and referral if required, and clear advice that SUDI protection always requires a sober caregiver.

**Table 2:** Testing the revised Safe Sleep Calculator model with the inclusion and exclusion of bedsharing and interactions with infant age, sleep position, smoking, alcohol and drugs on the new dataset from the New Zealand Nationwide Case–Control Study (NZ NWCCS) study data and the Safe Sleep Calculator pilot population data (AUC area under the curve, Odds Ratio OR).

	NZ NWCCS 2012–15			Safe Sleep Calculator pilot population	
	AUC	Sensitivity-cases identified (%)	Ideal fit for higher-risk cut-off	AUC	Infant population identified at high risk (%)
Carpenter Model Algorithm Includes bedsharing interactions with; infant age, sleep position, smoking, alcohol, and drug use	70.1	81.9	0.4/1000	87.8	20.9
Scenario testing 1–4					
1. Full revised Safe Sleep Calculator model algorithm with bedsharing interactions with; infant age, sleep position and smoking Alcohol, cannabis^a^ and other drugs have no interaction with bedsharing and OR each set at 2.0	78.5	79.5	0.3/1000	85.0	20.9
2. Remove age and age interactions from 1	77.7	79.3	0.3/1000	86.0	21.3
3. Scenario as in 2. and remove bedsharing and sleep position interaction Set alcohol OR 2.0, cannabis^a^ and other drugs each at OR at 2.0 with no interaction with bedsharing	74.2	83.0	0.3/1000	82.9	23.7
4. Scenario as in 2 and remove bedsharing interactions with and sleep position and drugs Set cannabis^a^ OR 2.0 and other drugs OR 2.0, alcohol set at OR 2.0 non-bedsharing or OR 4.0 if bedsharing	74.9	83.0	0.3/1000	88.9	19.1
	Brier score^b^ 0.18			Brier score^b^ 0.08	

^a^IPD meta-analysis and NZ pilot population data do not include separate data for cannabis vs other illicit drug use. Therefore the stated performance in the table does not account for instances of cannabis as well as other illicit drug use.

^b^The Brier score is a measure of predictive ability and here it is applied based on the binary outcome of at higher risk or not at higher risk—a Brier score of 0 is perfect, 1 is imperfect.

Although case series identify the association of SUDI with illicit drug use, the relative contribution of illicit drug use on SUDI risk, like alcohol, is poorly reported in the literature. Kandall et. al 1991 concluded that maternal ‘hard’ drug use (methadone, heroin, cocaine, and combination) during pregnancy increased the relative risk of SIDS by up to 3.7 fold (95% CI 2.6–5.3), which was the relative risk for methadone use, the drug associated with the highest risk after adjusting for high-risk variables (ethnicity, teenage mother, parity, maternal smoking, low birth weight)^24^. Conversely, Klonoff-Cohen and Phung 2001 did not find a significant association between maternal illicit drug use and SUDI when adjusted for other risk factors^25^. In the New Zealand cot death study, more than weekly cannabis use was associated with a SIDS odds ratio of 2.23 (95% CI 1.39, 3.57) after adjusting for maternal tobacco use^26^. In the clinical use of the Safe Sleep Calculator, the high OR associated with drug use resulted in an extreme risk to an infant, especially if bedsharing, that is relatively immovable by introducing other protective behaviours, and clinically unhelpful in motivating other behaviour changes (Table 1). Because drug use is unlikely to be an isolated risk factor and it is also physiologically plausible that maternal drug use will have varied effects on a developing fetus and maternal capacity to care for her infant, we have chosen to attenuate the risk associated with drug use, separate cannabis from other illicit drugs assigning each an OR of 2.0 and removed the interaction with bedsharing. Reported drug use prompts the revised Safe Sleep Calculator to provide clear individualized advice on the need to always have a drug-free caregiver to provide SUDI protection.

Because of a relative lack of evidence to guide altering the drug and alcohol risks in the algorithm, and the clinical use imperative to attenuate the risks, we tested various options for revision of the model for alcohol and drug use on the performance of the algorithm using the New Zealand Nationwide SUDI Case–Control Study (2012–2015) and the pilot -population dataset from the Safe Sleep Calculator pilot (Table 2). After making other adjustments to the model by removing the age interaction and the sleep position and bedsharing interaction, we tested attenuating the odds ratios associated with alcohol and drug use and removing the interaction of drug use with bedsharing (Table 2). The resulting changes in the model increase the effect sizes of parental smoking and bedsharing on risk when attenuating the effect sizes for alcohol and drug, and this resulted in an improvement in the performance of the overall algorithm, as measured by the Area Under the Curve (AUC) and Brier score, and specificity, whilst the sensitivity remained the same.

### Parental tobacco smoking and bed-sharing

Maternal smoking at any stage of pregnancy and postnatally is considered ‘maternal smoking’ for generating a SUDI risk score. There is an additional risk if the father smokes or if both parents smoke and the evidence is strong internationally that all scenarios of parental smoking and bedsharing for sleep results in an increased risk for the infant^12^. Diverse family arrangements (e.g. same-sex couples) have not historically been reported in case–control studies, but we have responded to clinical feedback and incorporated diversity in parenting into the revised web tool and have assigned maternal smoking risk to the person who has carried the pregnancy and the other parent or significant caregiver is assigned the ‘paternal’ smoking risk.

**Figure 3:**
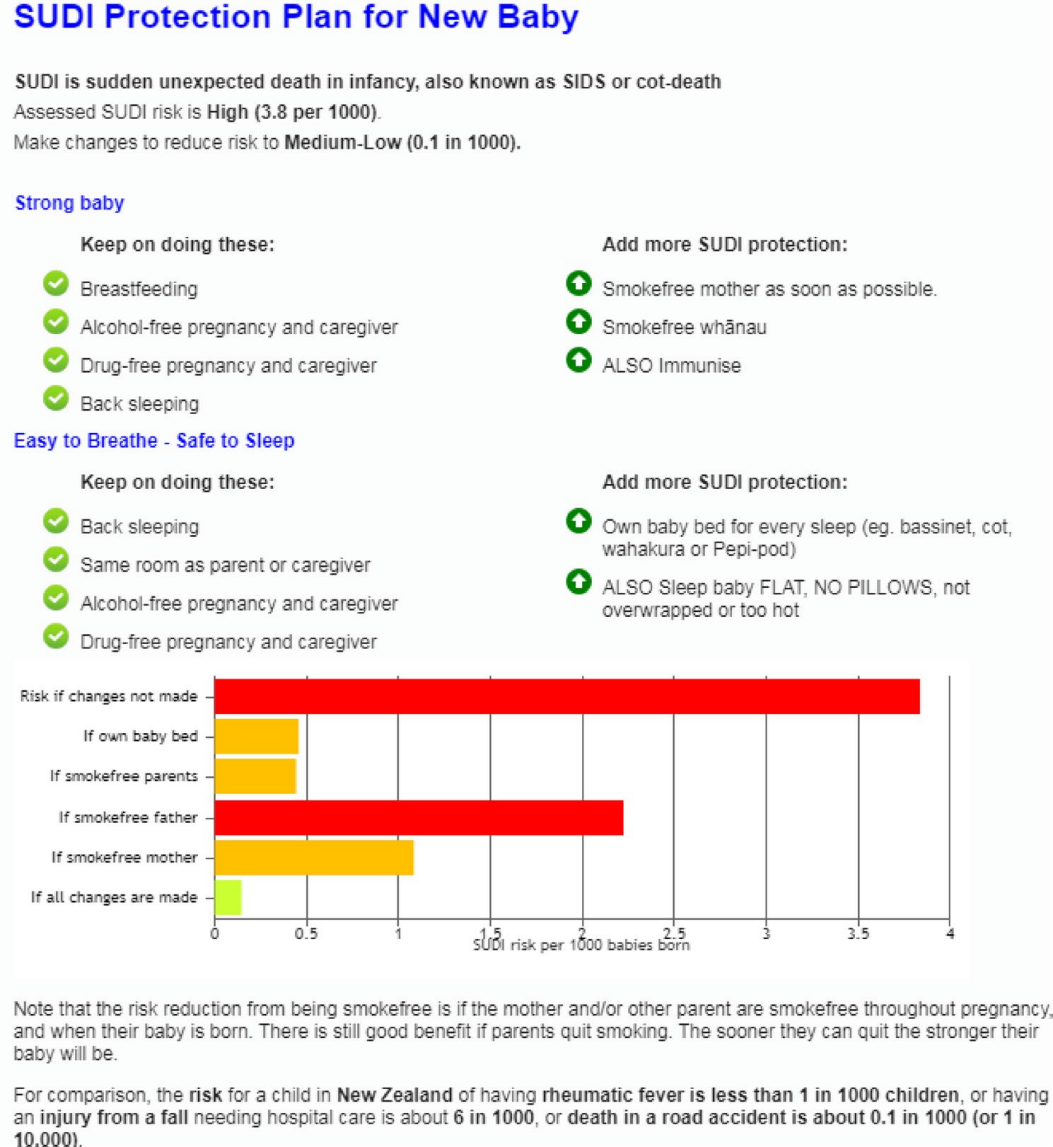
An example of the revised Safe Sleep Calculator result for a 3400 g female infant, third baby, mother and father smoke, mother does not drink alcohol or use drugs and her baby sleeps on her back and bedshares with her mother.

**Figure 4:**
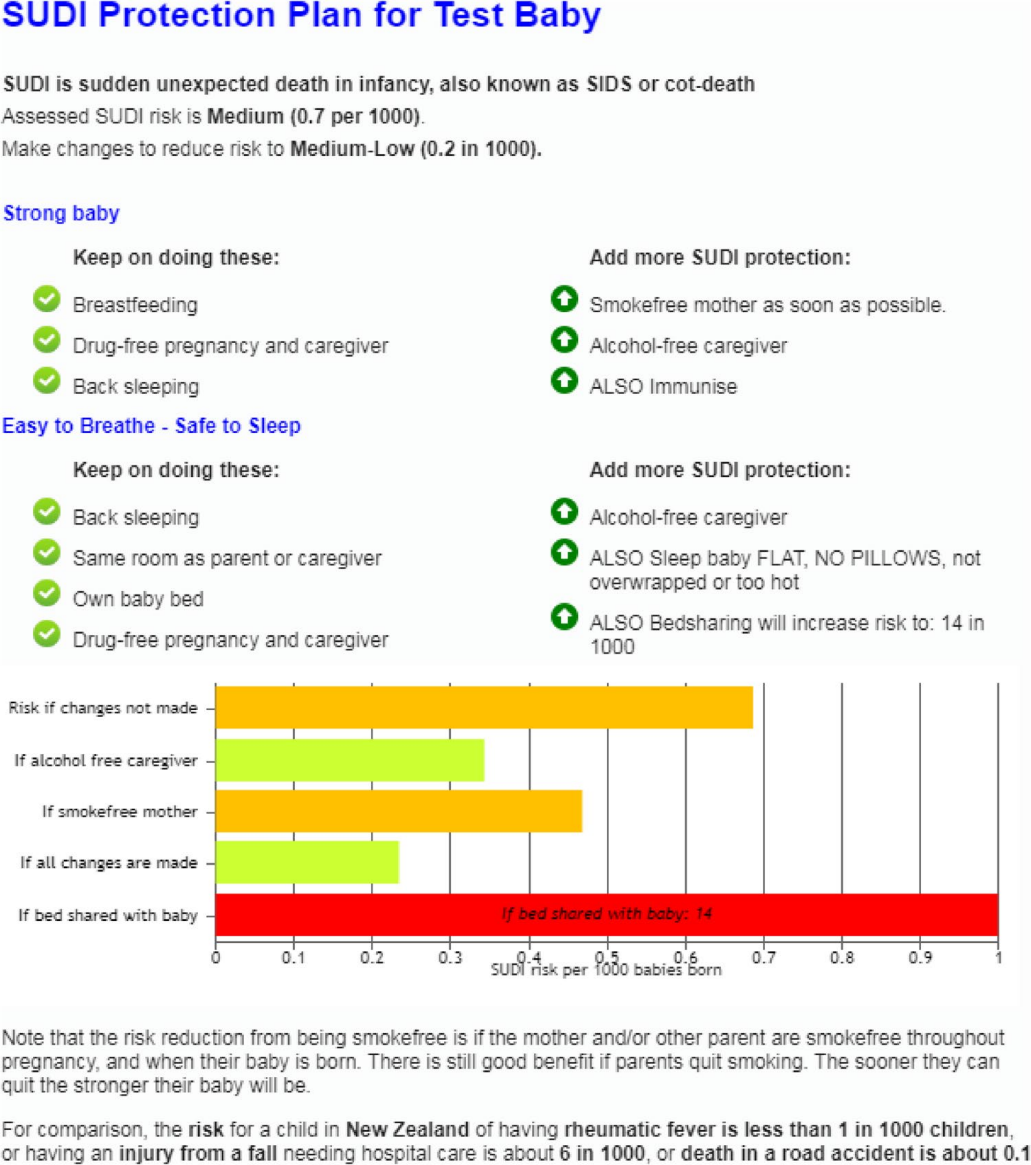
An example of the revised Safe Sleep Calculator result output for a 2980 g female infant, first baby for a 19-year-old mother who smokes, drinks two standard drinks 2–3 nights per week and sleeps her baby on their back in a cot. The result also shows the risk if she did bedshare. Note the difficulty showing the increased risk (14 per 1000) on the chart with bedsharing.

Infant sleep environments typically vary from sleep to sleep and for some families, bedsharing is usual practice, and for others, it is not planned but occurs, for example, as a response to an unsettled baby^27^. Māori and Pacific Peoples parents report that they may not admit to bedsharing with their baby because they fear being judged for this behaviour^15^. Through clinical use of the tool, it became clear that the result should take account of the answers given about bedsharing during the assessment, but irrespective of the result should also present the ‘what if’ risk for bedsharing. Figure 3. shows an example of an assessment when the mother does report bedsharing with the infant and Fig. 4. Shows the what-if bedsharing scenario.

### Presenting risk

When the original Safe Sleep Calculator tool was piloted, there were two main concerns with the resulting score; it was recognized that it was possible to enter variables such that the Safe Sleep Calculator returned an absolute risk of 1 in 1, and secondly, that the result appeared unrealistically accurate. While no clinical use episode returned a 1 in 1 result, the feedback from practitioners was that it was sufficient to group extreme high-risk into a greater than 1-in-50 (20-in-1000) risk category to motivate them as clinicians to urgently work with parents and families to address SUDI protection care. To address the concerns around the accuracy of absolute risk the revised Safe Sleep Calculator web tool is now coded to round the result and show it to one decimal point.

Clinicians also fed back that the presentation of risk was not visually impactful or understood by parents. The revised web tool displays risk and risk reduction on a chart, but there is still a challenge introduced by the ‘what-if bedsharing’ which is difficult to show accurately alongside the other contributing risks because its contribution is very high if there is maternal smoking (Fig. 4).

### Ethnicity

The Carpenter algorithm contained a variable labelled ‘white’/’non-white’ and attributed risk to non-white ethnicity. In the New Zealand context, the Māori and Pacific Peoples infant populations experience a significantly higher rate of SUDI than other populations, thus the ‘non-white risk was assigned to Māori and Pacific Peoples ethnicity. During the piloting of the Safe Sleep Calculator, the rationale for using ethnicity as a marker of risk was challenged by Māori practitioners because the ethnicity-specific label of being at-risk is stigmatizing and incorrect. In the New Zealand context Mitchell et. al. 1993 and Galland et. al. have shown that after adjusting for variables such as socioeconomic disadvantage and maternal education, Māori ethnicity per se is not a predictor of SUDI risk^28,29^. Kroll et.al. looked at ethnic-specific rates of SUDI in births in England and Wales 2006–2012 and showed that there was a substantial ethnic disparity between populations with Indian, Bangladeshi, Pakistani, White Non-British and Black African infants who had the lowest rates, British white infants in the intermediate rates, and Mixed Black-African-White, Mixed Black-Caribbean-White, Black Caribbean infant populations who had the highest rates, and they speculated that the differences may relate to ethnic-specific infant care practices^30^. Ethnicity has been removed from the New Zealand Safe Sleep Calculator risk assessment. Ethnicity is now factored in the prioritisation, after SUDI assessment for allocating additional SUDI prevention wrap-around support to address the disparity in SUDI rates between ethnic groups. We recommend defining high priority populations for SUDI prevention and applying the ethnicity variable for prioritisation purposes rather than risk scoring.

### External validation of the Safe Sleep Calculator algorithm for SUDI risk assessment

**Figure 5:**
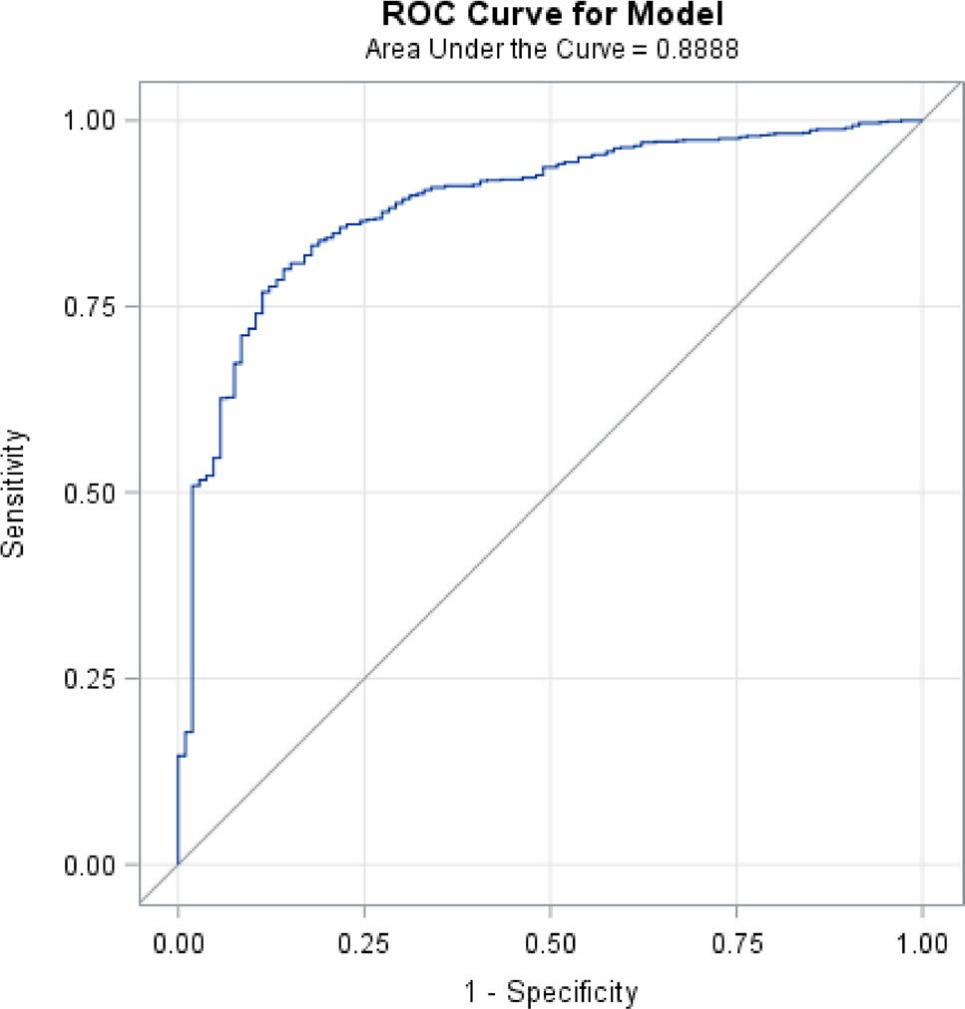
The Receiver Operator Characteristic (ROC) Curve for the revised Safe Sleep Calculator algorithm model applied to the New Zealand pilot population dataset.

To validate and assess the predictive value of the revised Safe Sleep Calculator algorithm in the New Zealand context we have applied the algorithm to a contemporary dataset not used in the Carpenter et. al. meta-analysis; the recent New Zealand SUDI Nationwide Case–Control Study^31^. This was a prospective study of all (n = 137) SUDI deaths occurring in New Zealand from March 2012 until February 2015. Interviews were completed for 133 (97%) of cases and 258 of controls (40% of the controls invited to participate). The controls for this study were frequency-matched to the distribution of SUDI cases for the four years preceding the start of the case–control study for infant age, obstetric hospital, and ethnicity. The control group was more closely matched to the cases than previous case–control studies, and therefore do not provide a representative sample of the whole birth population. The data generated by the Safe Sleep Calculator pilot in primary care from 966 individual assessments has provided a population infant dataset with similarity to the New Zealand maternal and infant population data as follows; Māori 29% vs 25%, Pacific Peoples 5% vs 10% median maternal age at the time of birth 29 vs 30 years, average birthweight 3463 g vs 3410 g, breastfeeding 79% at six weeks after birth vs 80% 2 weeks after birth, and maternal smoking in pregnancy population 17% vs 13%^32^. The performance of the revised Safe Sleep Calculator algorithm model is described in Table 2, scenario 4, and the receiver operating characteristic (ROC) curve for performance in the New Zealand pilot study population is shown in Fig. 5, with an AUC of 0.89 and a Brier score of 0.08. The ideal cut-off level for defining the higher-risk group from the lower-risk group is 0.3 per 1000 or more risk. The sensitivity of the algorithm to predict SUDI cases is 83.0% in the ‘at higher risk’ group, and the specificity in the New Zealand infant population context is 80.9%. In the New Zealand context, more intensive support for 310 infants (the number needed to treat) in the higher risk group has the potential to prevent one SUDI death, and intensive support will likely result in wellbeing benefits in addition to SUDI prevention such as increased breastfeeding, and reduced exposure to smoking, drugs, and alcohol.

## Discussion

This study has used a mixed-methods translational research design, to pilot, refine and validate a SUDI risk assessment algorithm embedded within the Safe Sleep Calculator clinical assessment tool used at the infant 6-week assessment in general practice primary care in New Zealand. This design has enabled the examination of each variable in the original Carpenter algorithm from a clinical use perspective, and its relative contribution of risk, to achieve a balance between sensitivity of the assessment tool and appropriateness of presentation for clinical use in the revised Safe Sleep Calculator. The Safe Sleep Calculator moves away from the provision of a standard set of SUDI prevention messages at a broad population level to a more individualized discussion about specific care needed to protect ‘this baby’. The World Health Organisation describes a one-on-one discussion to be a more trusted form of communication to make health messages accessible and actionable^33^. A strong motivation for the SUDI risk assessment algorithm was to support clinicians to provide evidence-based advice, particularly about bedsharing because of the potential conflict between cultural and family bedsharing norms, bedsharing in response to unsettled infants and maternal exhaustion, and the expectations by health care professionals of adherence to non-bedsharing SUDI prevention advice^27,34^. This is particularly important for population groups that have a higher prevalence of other factors creating risk for SUDI which elevates risk in a bedsharing situation. The Safe Sleep Calculator has achieved the aim of enabling an objective conversation about the risks and benefits of infant care practices.

The strength of both the Carpenter and Safe Sleep Calculator algorithm is that they use adjusted odds ratios from a large multi-national IPD dataset for many variables^12^. This is distinctive from early risk assessment scoring systems developed from single population data achieving sensitivities for predicting SIDS between 41 and 71%, in the highest 20% risk population, but these did not perform as well in other populations^9,35^. The most sensitive scoring system, the Sheffield Multi-stage scoring system, contained 17 variables including information from the first postnatal month, but in hindsight, it is clear that this and other scoring systems were hampered by variables that are no longer considered direct predictors of SUDI/SIDS risk^5^. The SUDI/SIDS literature now supports a remarkable consistency of adjusted odds ratios for known and well-established epidemiological risk factors, despite the difference in the prevalence of risk factors for populations in different parts of the world^12^. When baseline population risk is factored in it is expected that the Safe Sleep Calculator will perform well as a risk assessment tool irrespective of locality and the next step will be to test the algorithm in other populations.

SUDI risk is modifiable in the antenatal period and practitioners identified this during the pilot and recommend the Safe Sleep Calculator be used earlier than the six-week check, preferably during pregnancy or the first week of life. In 2006 Smith and White developed an antenatal SUDI assessment algorithm based on six obstetric characteristics to stratify risk which performed well predicting 72% of cases in the top 20% of predicted risk^18^. However, this algorithm was limited because it did not account for the effects of modifiable risks, nor the important interactions between factors such as maternal smoking in pregnancy and subsequent bedsharing with her baby^18,31^. We also considered a separate truncated antenatal algorithm, with similar performance, but recommend using the full postnatal Safe Sleep Calculator by assigning low-risk values for birthweight and sex of infant when assessing before birth and asking about intended infant care practices. We did this because the greatest benefit as a clinical tool was gained by asking about behaviours that are changeable and which may require additional support. The downside is that the SUDI assessment needs to be repeated at birth because a low birthweight can greatly impact risk and infants not previously identified as being at higher risk may otherwise be missed^12^.

A limitation of the Carpenter et. al. meta-analysis was the imputation required for IPD for historical case control studies, in particular for maternal alcohol and drug use which may have impacted in the development of the original and revised algorithms^12^. However, Moons et al. state that imputation can be a useful tool to include data from individuals which would otherwise be excluded and thereby unduly bias risk prediction models^36^. When the Carpenter algorithm was revised, modifications were made to the variable odds ratios, limits have been set to the extremes of variables, and some interactions were removed, and this may create concern about how this affects the validity of the Safe Sleep Calculator output. In a clinical pilot, it became clear that calculating extremely high risks was distressing and could be counterproductive to providing families with the opportunity to make changes that would promote SUDI protection for their baby. In other instances, such as sleep position and bedsharing, there was concern about conflicting and confusing messaging. Nevertheless, despite the concerns about limitations on the original data and these modifications for clinical use reasons, the revised Safe Sleep Calculator algorithm improved the sensitivity and specificity compared with the Carpenter algorithm as shown in Table 2 when tested in a new recent case–control data set and pilot population data. We hypothesise that this is because of the clustering of risk factors in babies at higher risk of SUDI, for example mothers who use drugs are also very likely to smoke during pregnancy. It is the magnitude of the effect and the interactions which are important and lack of precision of the odds ratio for maternal alcohol and drug does not unduly hinder the model. Ongoing use of the Safe Sleep Calculator coupled with New Zealand mortality data and observational data from routinely collected health information will allow the underlying algorithm to be further refined.

Showing risk in a meaningful way for both parents and clinicians has been a particular challenge. It is reasonable to argue that the risk estimates for some variables are optimistically precise in the model whereas for others they are not, such as maternal alcohol and drug use. The SUDI risk assessment output in the webtool suggests more precision than is due. However, it does help to contextualise the level of risk rather than using broad categories and can help to demonstrate benefit of risk reduction within a risk category. Further work is required to confirm risk estimates and to present risk assessment in a meaningful way. It is important to note that the external validation of the model in a contemporary dataset shows that the risk estimates used in the original algorithm based on older data remain valid and that the risk factors for SUDI are unchanged through the passage of time.

The Safe Sleep Calculator intends to provide one avenue to close the disparity in SUDI rates between high and low-risk populations. We believe it can do this in two ways; firstly, by providing an individually tailored objective SUDI risk assessment and specific evidence-informed prevention advice for families, and secondly by prioritizing the allocation of resources to infants at higher risk and their families for more intensive SUDI prevention care. Validation of the Safe Sleep Calculator in the pilot study New Zealand population dataset indicates that 19.1% of the infant population will be at higher risk, and this will identify 83.0% of future SUDI cases (Table 2 scenario 4). Some SUDI risk is relatively easily modifiable and at low-cost, such as back sleeping, breastfeeding, or baby sleeping in a suitable baby bed. However, smoking, alcohol, and drug use, and poor housing conditions, and overcrowding affecting safe sleep options for babies, are more complex to overcome because they often occur in a family or household context, and often because of poverty. Interventions for SUDI prevention need to support capacity, capability, and strong motivation to carry out the SUDI protective behaviour, which may require a wide range of support. The addition of SUDI scoring systems, with targeted interventions for families with babies at higher risk, in Sheffield, England, and West Virginia, USA, were both reported to have been associated with a reduction in SUDI/SIDS in the higher risk groups suggesting that the investment by health and social services is worthwhile^11,37^. The research team is now focussing on developing the Safe Sleep Calculator web-form as an assessment tool and also a platform for generating and tracking referrals for community-based wrap around SUDI prevention support in South Auckland, New Zealand, which has a culturally and ethnically diverse population with significant socioeconomic disadvantage and a high rate of SUDI^38^.

A limitation in the clinical use of the Safe Sleep Calculator is the risk of inaccurate assessment because parents may not disclose their normal behaviours for fear of being judged, and a higher risk result may cause anxiety and potential stigma for families. However, Māori and Pacific parents reported a strong desire to know if their baby was at increased risk and to avoid the potentially negative experience of it being used clinically, they wanted the opportunity to access the Safe Sleep Calculator themselves^15^. Consequently, it is important the Safe Sleep Calculator and the resulting information is delivered with care and respect to cultural and family norms and using a health literacy framework. A publicly accessible version of the Safe Sleep Calculator is being planned as an adjunct to the clinical use of the Safe Sleep Calculator tool^15^.

The Safe Sleep Calculator algorithm fulfils most of the criteria for a good screening tool; it screens for an uncommon occurrence that causes a large proportion of post-neonatal mortality, which is undetectable by other means and has effective prevention through supported behaviour change. While the use of the algorithm itself has not yet been proven to reduce SUDI deaths, the output of the clinical tool is evidence-based, and if protective behaviours are implemented, would be expected to reduce SUDI deaths. Interventions for babies (and their families) at higher risk are all non-invasive and are either neutral or have other beneficial health outcomes for babies, their mothers, and families. The Safe Sleep Calculator does not produce purely a binary result of ‘at higher risk’ or ‘at lower risk’, rather individualized recommendations—which means that it is not purely a screening test. However, using the Safe Sleep Calculator does have good sensitivity and specificity to determine the mothers and babies at the highest need of further evaluation and support for SUDI prevention. The negative predictive value of a lower risk result is 99.9% so there is no benefit to providing additional SUDI protection resources to the lower risk group. In the New Zealand context, the number needed to treat in the higher risk group is 310 to prevent one SUDI death at a background rate of 0.73 per 1000 live births^16^. The electronic web tool has transformed the algorithm into a usable clinical tool for integrating into the workflow at a comparatively low cost. Research is currently underway to evaluate the effectiveness of using the Safe Sleep Calculator for assessment of all babies born in South Auckland New Zealand, to determine whether adding a structured SUDI assessment and supportive care for families with babies at higher risk results in a reduction in the prevalence of modifiable SUDI risk factors in the population at higher risk.

## Summary

This paper presents the development of the Safe Sleep Calculator, a SUDI risk assessment algorithm presented in a clinical tool. A mixed-methods translational research approach was used to revise and validate the Safe Sleep Calculator algorithm for use in the clinical setting. Systematic use of the Safe Sleep Calculator can enable the opportunity for universal, but individually tailored SUDI protection advice, and concentration of SUDI prevention support to 19% of the New Zealand infant population at highest risk of SUDI, including the 83% of infants who would otherwise succumb to SUDI.

## Supplementary Information

Supplementary Information.
